# Influence of Light Exposure Time on the Vat Photopolymerization of Methacrylated PVA Microneedles

**DOI:** 10.1021/acsomega.6c04499

**Published:** 2026-07-14

**Authors:** Kaan Danis, Sule Ilgar, Shhd Saraj, Murat Topuzogullari, Oguzhan Gunduz, Cem Bulent Ustundag

**Affiliations:** † Department of Bioengineering, Faculty of Chemical and Metallurgical Engineering, Yildiz Technical University, Istanbul 34210, Turkey; ‡ Health Biotechnology Center for Excellence Joint Practice and Research (SABIOTEK), 52999 Yildiz Technical University, Istanbul 34349, Turkey; § Nanotechnology and Biomaterials Research Group, Department of Metallurgical and Materials Engineering, Faculty of Technology, 52982 Marmara University, Istanbul 34854, Turkey

## Abstract

Microneedles (MNs) provide a minimally invasive substitute for hypodermic injections, making them a revolutionary breakthrough in transdermal medication delivery. However, high prices and geometric limitations frequently restrict traditional fabrication techniques like micromolding. This work investigates the use of high-precision vat photopolymerization 3D printing to create hydrogel-forming microneedle arrays from poly­(vinyl alcohol) (PVA). By modification of PVA with methacrylic anhydride to methacrylated PVA (MPVA) and using lithium phenyl-2,4,6-trimethylbenzoylphosphinate (LAP) as a biocompatible photoinitiator, we created a photo-cross-linkable bioink. This study examined the effects of three different UV light exposure periods (30, 50, and 70 s) on the mechanical integrity, thermal stability, and morphological fidelity of the MPVA MNs in order to optimize the photopolymerization kinetics. The effective grafting of methacrylate groups onto the PVA backbone was verified by chemical analysis using FT-IR and NMR spectroscopies. A crucial trade-off was discovered by mechanical compression testing and scanning electron microscopy (SEM): while short exposure intervals (30 s) produced sharp but mechanically brittle structures, lengthy exposure times (70 s) produced strong but geometrically dull needles. The best processing window was found to be 50 s, which produced microneedles with enough mechanical stiffness. These results were further supported by differential scanning calorimetry (DSC), which demonstrated that longer exposure times improve the cross-linking density and heat stability of the polymer network. Parafilm-based insertion testing confirmed the functional penetration capability of MPVA-50 arrays, validating the optimized exposure condition as suitable for transdermal application These findings show that Vat photopolymerization printing of MPVA is a reliable, scalable, and adjustable technique for creating transdermal delivery systems.

## Graphical abstract

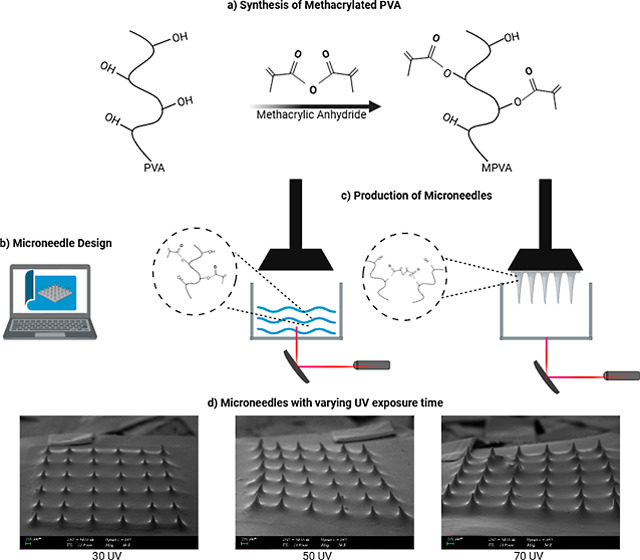

## 1 Introduction

3D tissue engineering has increasingly encompassed the development of microneedles, utilizing advanced additive manufacturing strategies to create precise, biofunctional scaffolds. Transdermal drug delivery systems, including microneedle-based patch platforms, are designed to transport therapeutically effective doses of drugs across the skin barrier.^1^ With advantages such as avoiding first-pass hepatic metabolism, prolonged therapeutic release, and increased patient compliance, the transdermal route has long been acknowledged as a desirable substitute for oral and parenteral drug delivery. However, the stratum corneum (SC), the skin’s outermost layer, severely restricts the effectiveness of transdermal administration. The SC limits the passive diffusion of most pharmaceutical drugs, especially those with high molecular weights or hydrophilic qualities, by acting as a strong barrier of keratinized corneocytes and lipids.^2^ Microneedles (MNs) are a revolutionary technology that can bridge the gap between the effectiveness of hypodermic injections and the convenience of transdermal patches. In order to mechanically puncture the SC and form temporary microchannels into the viable epidermis and dermis, MNs are micron-scale projections, usually 25 to 2000 μm in height.^3^ MNs fall into five main groups according to recent research: solid, coated, hollow, dissolving, and hydrogel-forming MNs. The ability of hydrogel-forming MNs to both swell for fluid extraction and distribute medications by diffusion without leaving polymer residues in the skin has made them popular.^4^

Laser ablation, lithography, micromolding, injection molding, and additive manufacturing are the main methods used in microneedle production, with micromolding and additive manufacturing being the most commonly preferred approaches. Although micromolding techniques played a major role in early MN research, these conventional methods are frequently limited by expensive master mold costs, design rigidity, and challenges in producing complicated geometries.^5^ As a result, the industry has quickly moved toward Additive Manufacturing (AM), and Vat Photopolymerization has become a better method for creating hydrogel MN.^6^ Laser assisted fabrication, regardless of array density or complexity, has parallel processing capacity which enables noticeably quicker print speeds.^7^ Laser assisted vat photopolymerization is used in the fabrication process, a liquid resin containing photo-cross-linkable polymers is selectively cured by a light source (405 nm LED) through the bottom of a transparent vat after a 3D Computer-Aided Design (CAD) model is cut into digital layers. The construction platform retracts after each exposure, allowing new resin to fill the space. This process is repeated until the entire array is created.^8^ This method is perfect for maximizing the sharpness and aspect ratio needed for successful skin penetration since it provides high resolution and the freedom to quickly develop designs.^9^

The choice of bioink materials is crucial to the successful creation of hydrogel MNs using vat photopolymerization. Gelatin methacryloyl (GelMA) and silk fibroin methacryloyl (SilMA) have been excessively used in this process to prepare novel transdermal drug delivery systems due to their excellent biocompatibility, biodegradability and tunable mechanical properties.^10–12^ Nevertheless, despite these advantages, challenges such as limited mechanical robustness and batch-to-batch variability remain. In contrast, studies exploring the utilization of synthetic yet biocompatible polymers in Vat photopolymerization based hydrogel MN production are comparatively limited, highlighting the need for further investigation. For this purpose, poly­(vinyl alcohol) (PVA) was chosen due to its high mechanical strength, biocompatibility, chemical stability, and adjustable hydrophilicity.^13^ However, PVA needs to be functionalized with methacrylate groups using methacrylic anhydride (MA) for radical-mediated photopolymerization in Vat photopolymerization. By adding pendant methacrylate groups to the PVA backbone, this modification enables the polymer chains to covalently cross-link when exposed to UV light.^14^

The exposure time, or how long each layer is lit under UV irradiation during the production process, is one of the most important factors affecting the MNs physical characteristics. The Beer–Lambert rule governs the polymerization kinetics, where the cure depth (*C*_*d*_) is logarithmically connected to the ratio of exposure energy (*E*) and the critical energy (*E*_*c*_) needed for gelation.^15^ Significant trade-offs between mechanical rigidity and microneedle quality result from variations in exposure time. Insufficient cross-linking from under-exposure produces flimsy, brittle needles that might not stick to the construction platform or buckle when they come into touch with skin.^10^ On the other hand, excessive exposure results in light scattering, where the resin cures past the desired limits. This enhances the MNs’ mechanical stiffness (Young’s modulus), but it also increases their tip radius, making the needles blunt and requiring too much force to penetrate the skin.^16^ Finding the ideal exposure duration is therefore a crucial step in striking a balance between geometric accuracy and structural stiffness.^17^

Building on this premise, we systematically investigated, for the first time, the role of light exposure time in the Vat photopolymerization fabrication of MPVA-based hydrogel microneedles. In this context, we comprehensively evaluated changes in mechanical strength, rheological behavior, and morphological characteristics as a function of exposure duration. Particular attention was paid to identifying fabrication parameters that enable the formation of mechanically robust, geometrically sharp, and structurally stable MN arrays capable of penetrating the stratum corneum without deformation or fracture. The study provides insights into optimizing MPVA-based hydrogel MN production, thereby contributing to the rational design of Vat photopolymerization fabricated hydrogel MN platforms for transdermal delivery applications.

## 2 Materials and Method

### 2.1 Materials

PVA (98–99% hydrolyzed, Mw: 31–50 kDa), MA, LAP and dialysis membrane (MWCO: 10 kDa) were from Sigma-Aldrich. Distilled water was used from Blulab (Blueaqua, Türkiye)

### 2.2 Methacrylation of PVA

The process of adding methacrylate groups to the PVA polymer chain was carried out according to a previous work.^18^ Ten wt % of PVA solution was prepared by dissolving PVA in distilled water under constant heating at 90 °C for 2 h, followed by cooling to room temperature. 37.5 μL of MA was added per 1 mL of the dissolved PVA solution. MA was added to the PVA solution dropwise for 1.5 h, while keeping the pH constant at 8 for this duration. The solution was then dialyzed against distilled water for 5 days and lyophilized. The collected MPVA was stored in +4 °C.

### 2.3 Chemical Analysis of the Materials

The resulting MPVA substitution degree was analyzed using NMR spectroscopy (Bruker AVANCE). D_2_O was used as solvent to dissolve MPVA in 10 mg/mL concentration. After acquiring H NMR spectra at room temperature at a frequency of 500 MHz, the degree of substitution was calculated using the eq 1:^18^

1 $$DS\left(\%\right)=\left(I_{vinyl}/2\right)/I_{backbone}$$

The molecular structure and chemical properties of the MN were investigated using FT-IR (Fourier Transform Infrared) spectroscopy (PerkinElmer spectrum 100, USA). Spectra were acquired within the range of 400–4000 cm^–1^ with a resolution of 4 cm^–1^.

### 2.4 Rheological Analysis of the MPVA Solution

Using an Anton Paar rheometer (Anton Paar, Austria), the rheological behavior of the MPVA solution was analyzed in order to assess its flow characteristics and compatibility for the Vat photopolymerization printing process. A parallel plate geometry (25 mm diameter) with a gap distance of 0.5 mm was used for all measurements which were carried out at 25 °C. Rotational viscosity tests were carried out by adjusting the shear rate from 0.1 to 100 s^–1^ in order to evaluate the bioink’s printability and liquid-state stability. Furthermore, an oscillatory frequency sweep test between 0.1 and 100 Hz at a fixed strain amplitude inside the linear viscoelastic area was used to assess the viscoelastic properties, namely the storage modulus (G′) and loss modulus (G″). Real-time photorheology was used to track the MPVA solutions photo-cross-linking activity in order to measure the gelation time. The Anton Paar rheometer with a photocuring accessory (glass bottom plate) to enable light irradiation was used for the measurements. To ensure the sample remained in the linear viscoelastic zone, an oscillatory time sweep was carried out at a constant frequency of 1 Hz and a strain amplitude of 1%. The resin was exposed to 405 nm light through the bottom plate following a short stabilization period in the dark. As a function of exposure time, the storage modulus (G′) and loss modulus (G″) were continually measured. The gelation point was defined as the crossover point where the storage modulus (G′) surpassed the loss modulus (G″), indicating the transition from a liquid sol to a solid gel network.

### 2.5 Vat Photopolymerization Printing

The microneedle design was adopted from a previous study.^8^ Briefly, SolidWorks 2020 was used to create CAD files of tapered microneedle arrays with needle heights expressed in micrometers. A solid 10 × 10 × 1 mm base with a 6 × 6 array was bonded to each microneedle, which was made to be 1500 μm broad at the base. The 3D bioprinting software of Chitubox was then used to transform the microneedle designs into.stl file format. The software sliced them using varied exposure intervals of 30, 50, and 70 s and the samples were designated as MPVA-30, MPVA-50, and MPVA-70, respectively.

Ten wt % MPVA solution was prepared by dissolving MPVA in distilled water at 85 °C under constant stirring, followed by cooling to room temperature. LAP was added to the solution at a concentration of 0.25%. The solution was then poured into the vat of the Anycubic printer (Anycubic Photon Mono M7 Shenzhen/China) and the designed MN platforms were printed. Printed MN samples were stored at +4 °C for future use.

### 2.6 Morphological Analysis of MPVA MN

SEM (EVA MA 10, ZEISS, USA) was used to analyze the size and surface morphology of the generated MNs. Prior to imaging, a sputter coating machine (Quorum SC7620, USA) applied a 120 s gold coating to the samples’ surface. SEM images were obtained at 50 X, 70 X, and 150 X magnifications under an accelerating voltage of 10 kV. ImageJ software was used to measure microneedle heights in accordance with previously outlined procedures.^9^

### 2.7 Thermal Characteristics of MPVA MN

A differential scanning calorimeter (Shimadzu, Japan) was used to thermally characterize the MN samples of MPVA-30, MPVA-50, and MPVA-70. Each sample, weighing about 2 mg, was sealed in an aluminum crucible and heated under air at a rate of 20 °C per minute from 25 to 300 °C.

### 2.8 Mechanical Properties of the MPVA MN

The mechanical characteristics of MPVA MNs were determined via compression tests. A compression testing apparatus (EZ-LX, Shimadzu Corporation, Japan) with a 5 kN ± 0.5% load cell at ambient temperature and a displacement resolution of 1 μm was used for the tests. Cylindrical hydrogels were prepared according to MN samples with a height of 2.5 mm and a diameter of 7.0 mm. Compression tests were conducted at a rate of 1 mm/min. Analysis was conducted in triplicate and standard deviation was given.

### 2.9 Penetration Analysis of the MPVA MN

The penetration abilities of MN were evaluated using parafilm technique.^10^ The parafilm, with each layer measuring approximately 127 μm in thickness, was folded into 6 layers. Three replicate arrays per condition (*n* = 3, 36 needles per array) were then inserted into the Parafilm stack by manual thumb pressure and subsequently removed. The layers were carefully separated one by one, and the number of holes in each layer were evaluated using an optical microscope (iScope IS.1152-PLi, Euromex, Arnhem, Netherlands) and values were reported as mean ± standard deviation across the three replicates.

## 3 Results and Discussion

In this study, methacrylation of PVA was carried out as the initial step in the fabrication of MPVA MNs. The chemical structure and degree of methacrylation were characterized using NMR and FTIR spectroscopy, followed by rheological analysis of the MPVA precursor solutions. Subsequently, MNs were fabricated from MPVA, and their chemical, morphological, thermal, and mechanical properties were systematically evaluated.

The successful synthesis of MPVA was confirmed NMR spectroscopy, which revealed distinctive peaks in ^1^H NMR spectrum (Figure 1a) confirming the grafting of methacrylate groups onto the PVA backbone. The vinyl protons (–C=CH_2_) of the methacrylate group are specifically responsible for the emergence of signals in the range of 5.70–6.00 ppm (pink strips in Figure 1a), indicating the esterification reaction between PVA’s hydroxyl groups and methacrylic anhydride was successful.^18^ The degree of substitution (DS) was derived from the integration ratio of the vinyl protons to the hydroxyl protons (between 4.00 and 3.90 ppm, green strip in Figure 1a) of the PVA backbone. The DS was found to be 3.2%, which is sufficient to promote photo-cross-linking throughout the Vat photopolymerization procedure.

**Figure 1:**
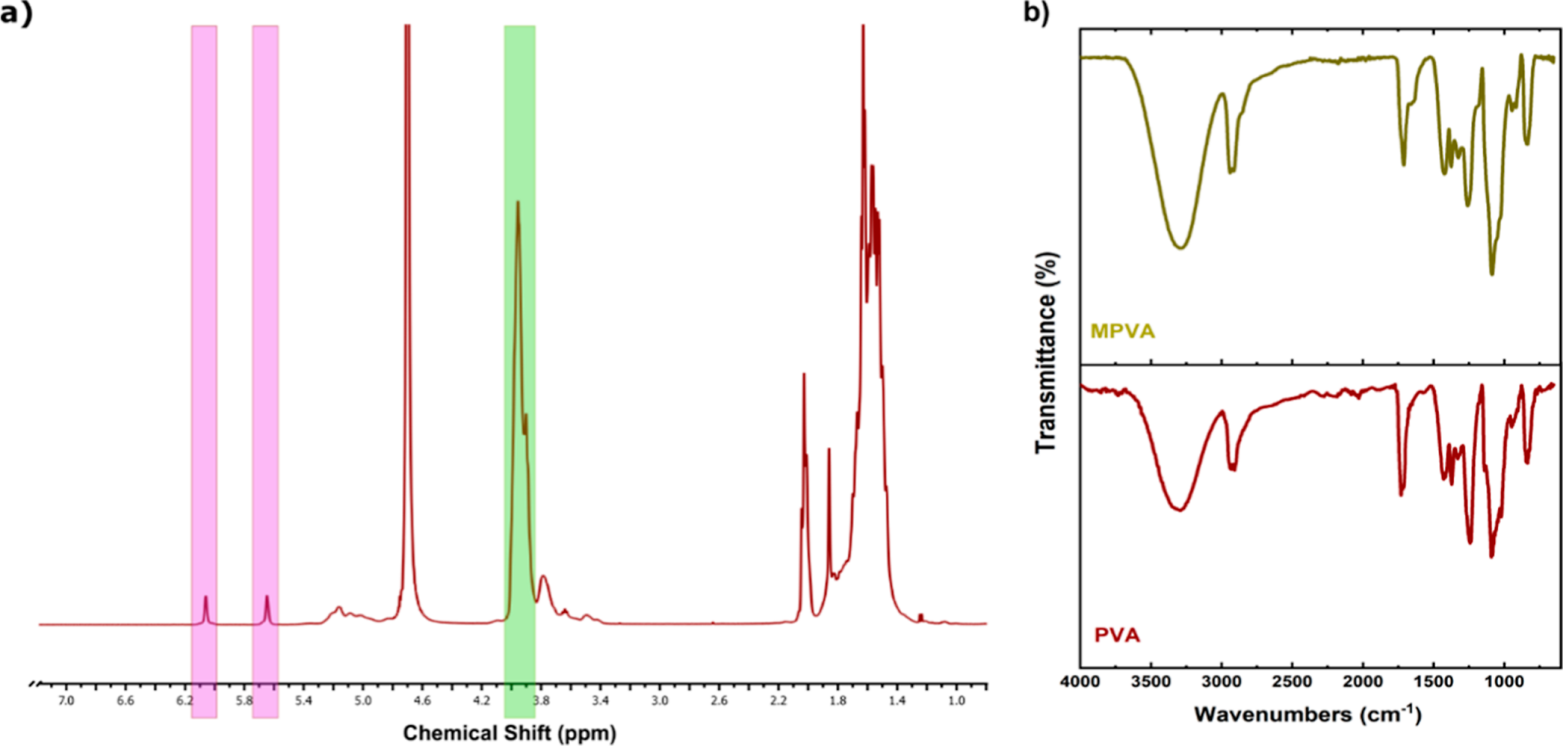
^1^H-NMR spectrum of MPVA (a). FTIR spectra of PVA and MPVA (b).

FT-IR spectrum (Figure 1b) offered further structural validation in addition to NMR spectrum. A broad strong band at 3200–3400 cm^–1^ in the spectra of unmodified PVA was indicative of O–H stretching vibrations. The C=O stretching vibration of the ester carbonyl group created during methacrylation is represented by a new, unique peak that emerged in the MPVA spectra at about 1720 cm^–1^. Furthermore, compared to pure PVA, the MPVA samples showed a decrease in the relative strength of the O–H band, which suggests that hydroxyl groups were consumed during the methacrylation reaction.^19^

In the printing process, the flow behavior of the resin directly affects the layer formation quality. Therefore, rheological analysis of MPVA solutions is critical for preparation of desired MNs. To guarantee printability, rheological behavior of the MPVA resin (10 wt %) was examined (Figure 2). The resin used in vat photopolymerization printing needs to have a viscosity that is both high enough to preserve stability during localized curing and low enough to reflow quickly and cover the build window during the lifting and retracting stages. Viscosity, frequency analysis and photorheology were analyzed, and the acquired results are given in Figure 2. The viscosity measurement showed a shear thinning behavior while the frequency analysis showed small differentiations with the addition of LAP. Photorheology result revealed a fast gelation at the 5 min mark.

**Figure 2:**
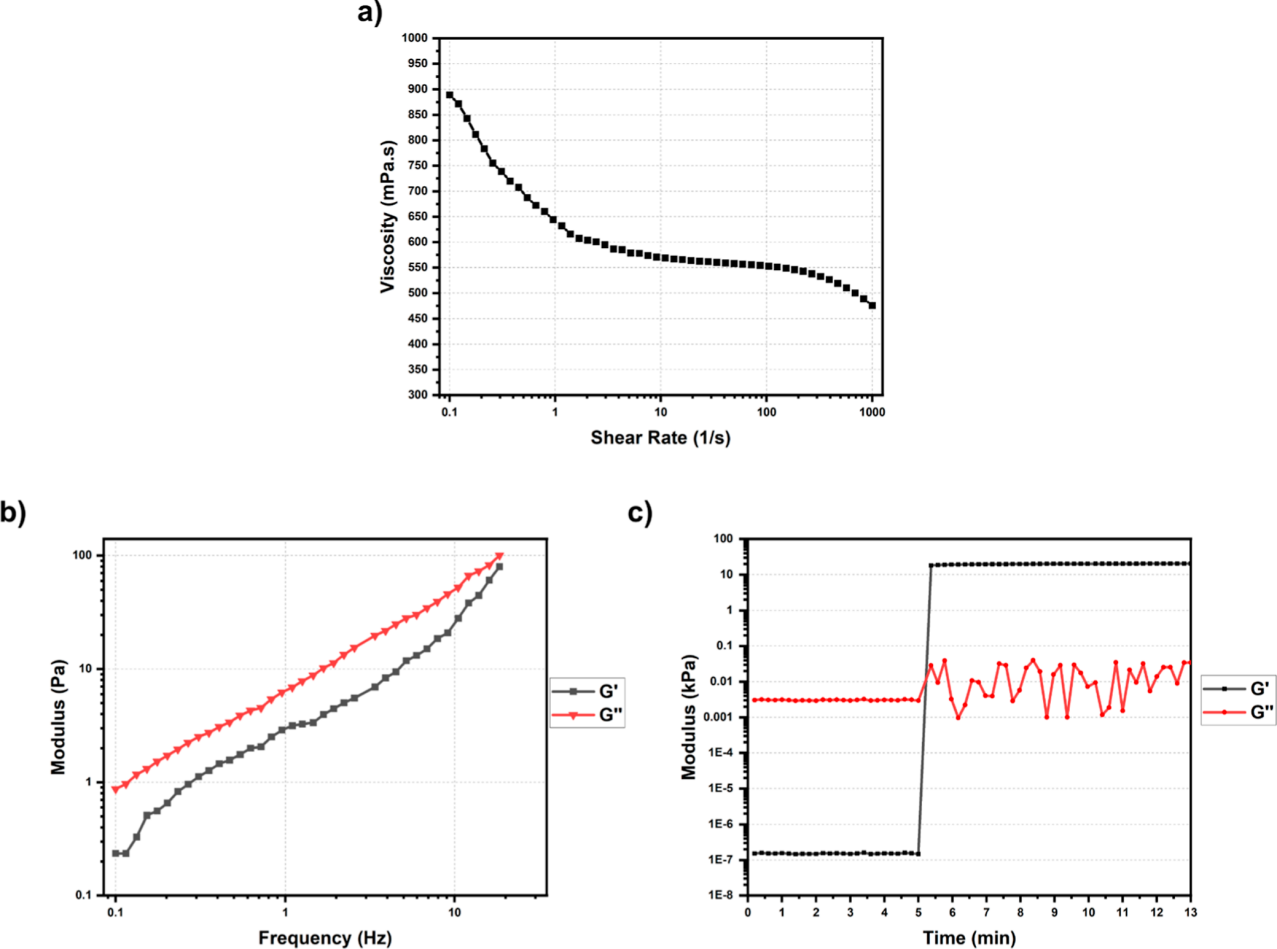
Rheological analysis of the MPVA precursor solution, showing viscosity as a function of shear rate (a), frequency sweep results (b), and gelation behavior during photopolymerization (c).

The shear thinning property (Figure 2a) of the solution allows the resin to flow easily during the movement of the printing platform while maintaining its form during UV exposure. To prevent the resin from gelling too soon in the vat, frequency sweep analysis (Figure 2b) verified that the resin behaves primarily as a viscous liquid (G″ > G′) before UV treatment.^20^ The “crossover” point (gelation point) observed in photorheology tests occurred at approximately 5 min, proving that MPVA has a sufficient polymerization rate within the printing time.^21^ The gelation time of approximately 5 min observed in photorheology reflects the bulk transition under relatively low-irradiance diffuse illumination conditions used in the rheometer setup. In the vat photopolymerization process, the high-intensity collimated LED array of the printer delivers a substantially higher energy dose per unit time, enabling complete per-layer curing within 30–70 s. The photorheology result therefore serves as a confirmation that the MPVA/LAP formulation does not undergo premature gelation in the resin vat and has a workable pot life compatible with the printing process, rather than a direct predictor of per-layer cure time. The potential of MPVA/LAP ink formulation in high-resolution vat photopolymerization manufacturing is confirmed by this rheological profile.

After confirmation of successful methacrylation of PVA and its suitability as a resin, we prepared MNs with MPVA under 30, 50, and 70 s of laser irradiation. Figure 3 exhibits the FTIR spectra of the produced MNs of MPVA-30, MPVA-50, and MPVA-70. The FTIR spectra of the samples remained consistent, indicating that the chemical structure of the polymer network is maintained regardless of the exposure time used during printing.

**Figure 3:**
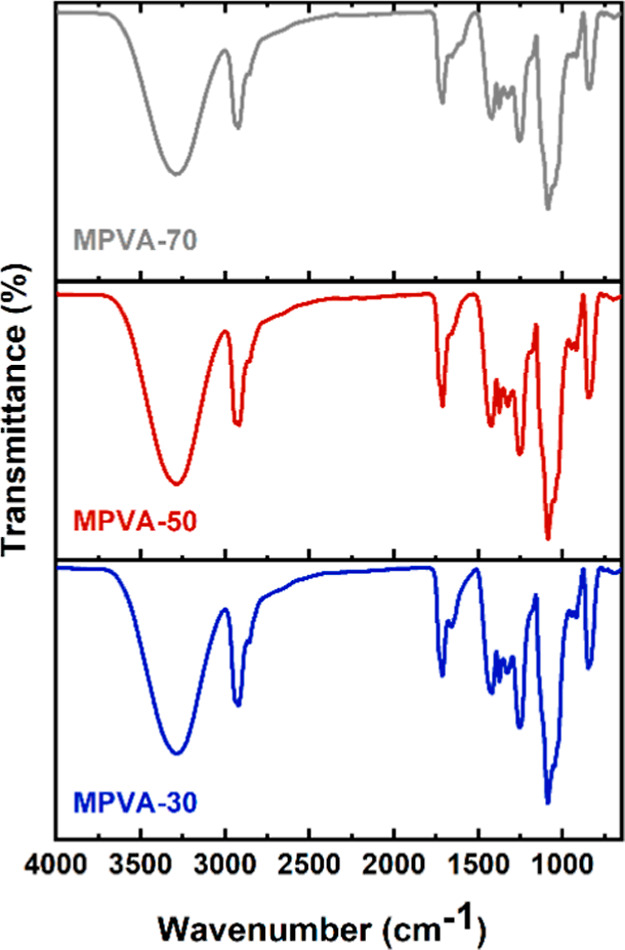
FTIR spectra of MNs of MPVA-30, MPVA-50 and MPVA-70.

SEM was used for imaging of MNs to confirm the MN formation and morphology of the prepared MNs. Figure 4 shows the SEM images of MPVA30, MPVA50 and MPVA70 acquired at different magnifications. SEM image of MPVA-30 revealed clearly defined sharp needle tips but under-curing leading to reduced needle height. The applied energy (ε) barely exceeded the critical threshold (ε). This resulted in ε being limited and the average needle height being measured at its lowest level of 319.093 ± 2.724 μm. The low energy input prevented sufficient cross-linking of the resin, causing the needles to remain sharp but under-cured. On the other hand, an overcuring phenomenon was seen in the MPVA-70 samples with needle heights reaching 396.366 ± 14.543 μm. The needles showed larger diameters and a noticeable blunting at the tips as a result of light scattering within the resin at high energy dosages. This is due to the fact that the Beer–Lambert law affects not only vertical curing but also the distribution of light within the resin. The high energy input caused light scattering and curing beyond the targeted geometric limits (“voxel bleed”). This situation led to the bluntness of the needle tips, an increase in their diameter, and a loss of needle sharpness. The effective tip radius is greatly increased by this tip blunting, and the insertion force needed to pierce the stratum corneum is known to increase exponentially.^22^ The MPVA-50 showed a smooth, continuous surface morphology with needle heights reaching 352.739 ± 3.643 μm. This height is adequate for penetration of the skins layer.^23^ There is an ideal window in which the resin cures precisely to the borders without experiencing blunting.

**Figure 4:**
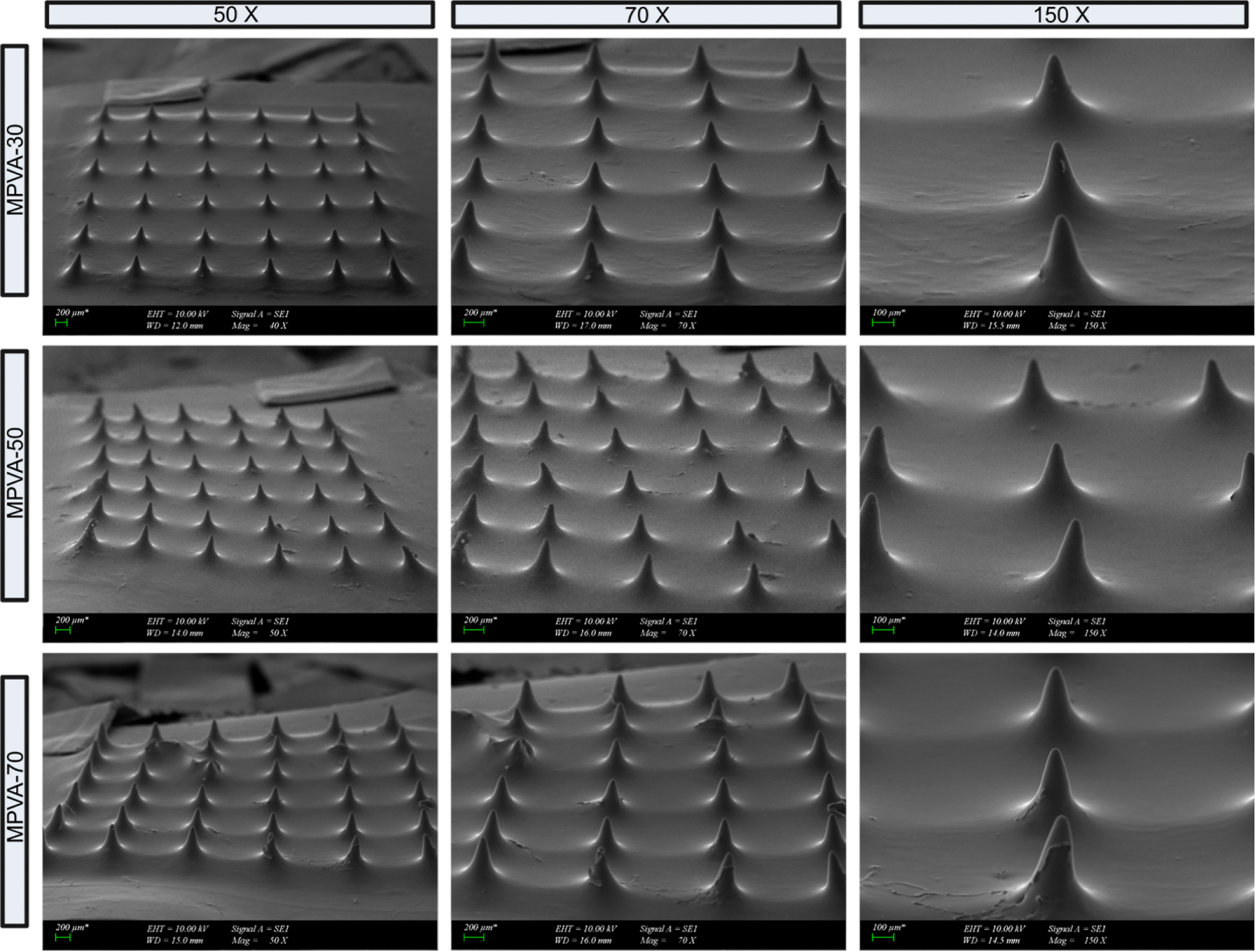
SEM images of the printed MNs of MPVA-30, MPVA-50 and MPVA-70 at magnifications of 50 X, 70 X and 150 X.

SEM analysis showed that the printed microneedles’ shape was significantly influenced by the exposure time per layer. The goal of the fabrication method was to create conical microneedles, but different photon energy dosages produced different geometrical results.

Sufficient mechanical strength is critical to ensure successful skin penetration of microneedles without structural failure. Mechanical behaviors of the produced MNs were analyzed and their compressive strength (MPa) and strain (%) values are given at Figure 5.

**Figure 5:**
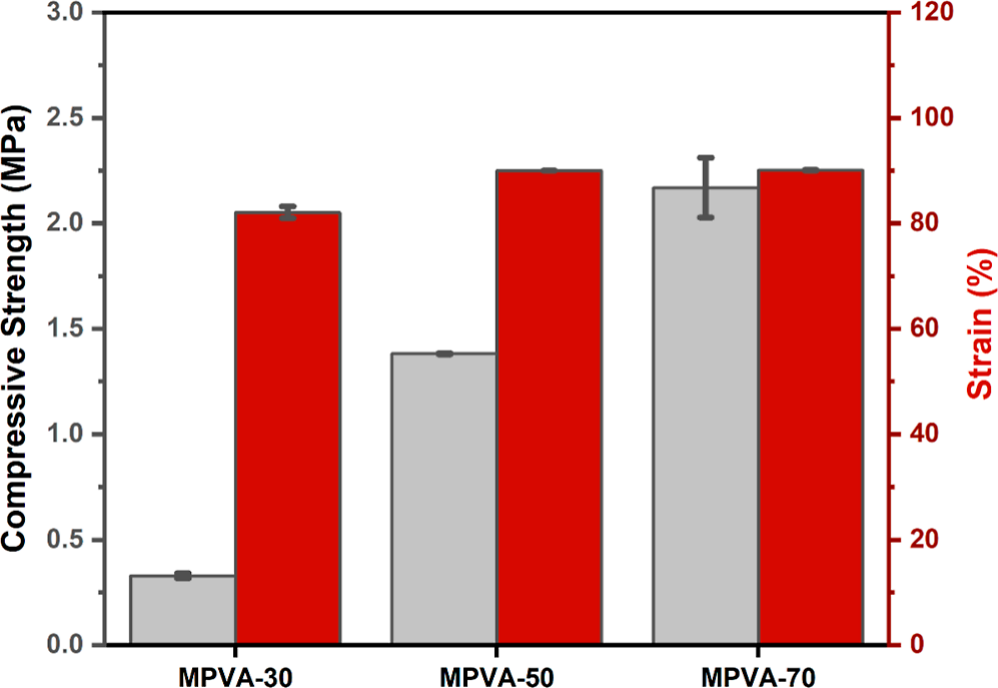
Compressive strength (gray columns from 0 to 2.5 MPa) and corresponding strain values (red columns from 80 to 90.5%) of the produced MNs.

MPVA-30 showed the lowest compressive strength of 0.328 ± 0.013 MPa. Low degree of conversion is probably the cause of the viscoelastic, rubbery behavior suggested by the stress–strain curve. Instead of piercing the skin, these needles are likely to buckle under axial load.^24^ The samples with the highest failure force and stiffness were MPVA-70 with compressive strength of 2.169 ± 0.142 MPa. A stiff, glassy network is produced as a result of the prolonged exposure duration, which increases the creation of covalent cross-links. However, the blunter tips of the MPVA-70 needles may counteract the advantage of greater stiffness by needing excessive force for insertion, as indicated by the SEM analysis.^25^ MPVA-50 showed mechanical qualities with compressive strength of 1.381 ± 0.003 MPa, strong enough to endure the normal insertion force of human skin (0.1 to 3.0 N per array) without buckling.^26^ Tip compression predominated over catastrophic base fracture as the failure mode.

The findings demonstrate the trade-off in vat photopolymerization printing: longer exposure times increase mechanical strength, which is advantageous, but they also reduce tip sharpness, which is disadvantageous. It should be noted that compression testing was conducted on bulk cylindrical hydrogel specimens prepared under the same UV exposure conditions as the printed arrays (MPVA-30, MPVA-50, and MPVA-70), rather than on the printed microneedle geometries directly. The results therefore reflect the intrinsic bulk stiffness and failure behavior of the cross-linked MPVA network, serving as a foundational mechanical profile required to accurately model and understand subsequent insertion mechanics.

DSC analysis was used to evaluate the thermal transitions of the MPVA MNs (Figure 6). The thermal properties of PVA and its derivatives are known to be affected by modifications that alter crystallinity, chain mobility, and cross-link density. In unmodified PVA, moisture loss often appears as a broad endothermic event at lower temperatures, followed by thermal transitions associated with melting or relaxation of crystalline domains.^27^ Methacrylation and subsequent UV cross-linking further influence these transitions by restricting chain movement and reducing crystalline ordering.^28^

**Figure 6:**
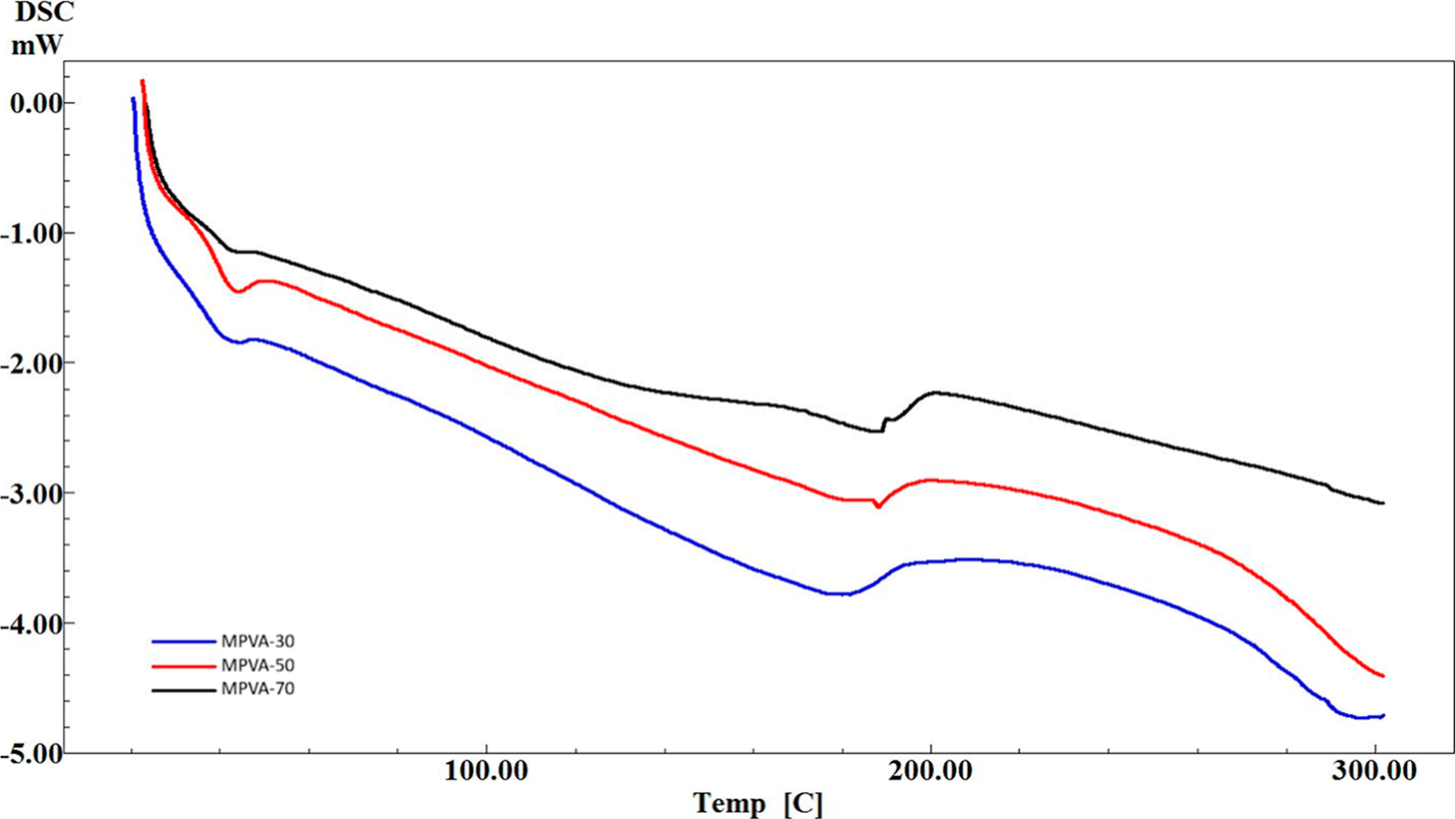
DSC curves of MPVA-30, MPVA-50 and MPVA-70 MNs.

For the MPVA-30 sample, the DSC trace showed a broad endothermic region between ∼50–80 °C attributable to moisture evaporation, which is typical for hydrophilic polymers like PVA due to bound water release.^27^ Thermal transitions and pronounced endothermic events observed at low temperatures in the range of 150–210 °C in 30 s samples suggest that the cross-link density in the polymer network is low^28^ and the polymer chains still have high mobility. This finding explains why MPVA-30 shows the lowest compressive strength in mechanical tests and exhibits “rubbery” viscoelastic behavior. The network structure is not “locked” enough to resist under mechanical load.

Increasing the exposure time to 70 s caused the thermal transitions to shift to 190–220 °C range and the peak intensities to decrease, suggesting the formation of a dense cross-linked network structure. Increased UV energy restricted chain mobility by enabling the formation of more covalent bonds between methacrylate groups. Similar behavior has been reported for methacrylated polymer systems, where higher UV exposure leads to more robust cross-linked networks and altered DSC behavior.^29^ This thermal stability is in complete agreement with the high Young’s modulus and “glassy” hard structure observed in mechanical tests. However, the “voxel bleed” (light scattering) and thickening at the needle tips detected in SEM images imply that this high cross-linking occurred at the expense of geometric precision.

The thermal profile of the MPVA-50 sample confirms that there is an ideal balance point between the unstable structure of MPVA-30 and the extremely rigid structure of MPVA-70. The fact that the thermal transitions are located in the midtemperature band shows that the material has a network structure that is stable enough not to be affected by body temperature during skin penetration, but flexible enough not to create brittleness. The thermal analysis provides data at the molecular level supporting superior mechanical suitability of MPVA-50.^30^

The progressive shift of thermal transitions and reduction in endothermic intensity from MPVA-30 to MPVA-70 support the conclusion that UV exposure time is a key determinant of the thermal stability and structural configuration of MPVA networks. These findings are consistent with the role of cross-linking in enhancing thermal resistance and mechanical properties of polymeric hydrogels, which is crucial for microneedle performance.^31^

To assess the insertion capability of the MNs, a parafilm penetration test was performed and results are given as holes created (%) to each parafilm layer in Figure 7. All three conditions achieved 100% penetration efficiency at layer 1 (∼127 μm), confirming that each sample produces sufficient mechanical force for initial skin surface penetration. However, the conditions diverged significantly at greater depths, revealing a clear exposure-time-dependent trend in penetration performance.

**Figure 7:**
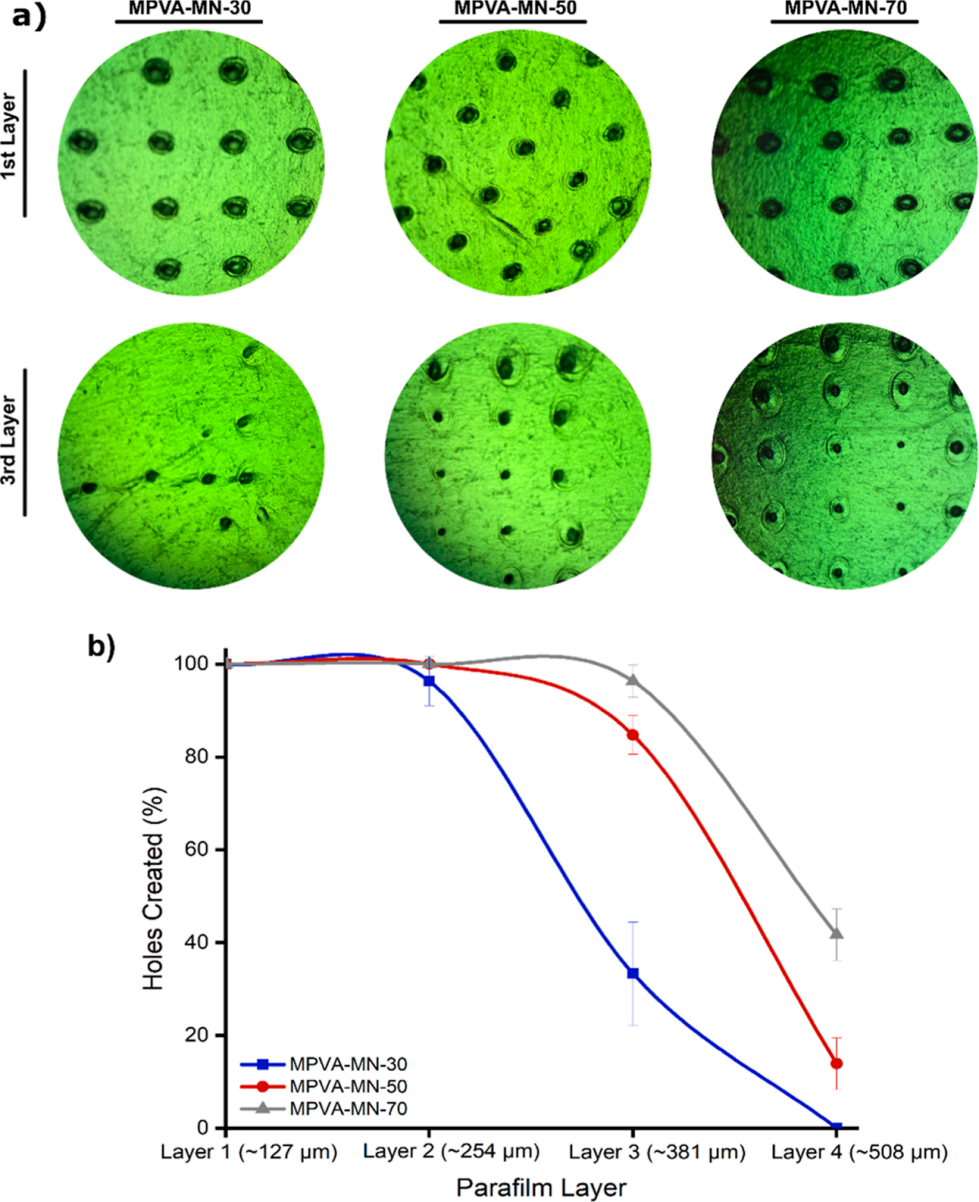
Microscope images exhibiting the penetration of the produced MNs into the parafilm layers (a) and puncture percentage of microneedles in parafilm layers as a function of the number of layers (b).

MPVA-30 showed a rapid decline in penetration efficiency with increasing depth, dropping to 96.3 ± 5.2% at layer 2, 33.3 ± 11.1% at layer 3, and reaching complete penetration failure (0%) at layer 4 (∼508 μm). This progressive loss of penetration capability is consistent with the low mechanical stiffness and rubbery viscoelastic behavior observed in compression testing, where under-cured needles are prone to buckling under sustained axial load rather than transmitting force to the tip.

MPVA-50 maintained complete penetration through layer 2 (100 ± 0%) and retained strong performance at layer 3 (84.7 ± 4.2%), with a drop to 13.9 ± 5.5% at layer 4. The relatively high standard deviation with increasing layers indicates that MPVA-50 needles are operating near their mechanical penetration limit at this depth, consistent with a needle height of 352.74 ± 3.64 μm relative to a cumulative parafilm depth of ∼508 μm. This behavior is attributed to viscoelastic deformation of the parafilm during application, which reduces the effective penetration distance at the needle tip, as previously reported in microneedle insertion studies.^26^ Nevertheless, MPVA-50 demonstrated consistent and reliable penetration within the therapeutically relevant depth range of the stratum corneum and viable epidermis (∼200–400 μm).

MPVA-70 exhibited the highest penetration depth overall, maintaining 96.3 ± 3.5% efficiency at layer 3 and retaining 41.7 ± 5.6% at layer 4. The superior penetration depth of MPVA-70 relative to MPVA-50 is consistent with its higher compressive stiffness, which enables greater axial force transmission even at increased depths. However, this mechanical advantage must be considered alongside the geometric hurdles identified by SEM analysis. The tip blunting caused by voxel bleed in MPVA-70 increases the required penetration initiation force and produces larger, less defined puncture profiles, which are suboptimal for a transdermal patch intended to create precise microchannels.^22^ No penetration was recorded beyond layer 4 for any sample.

A systematic evaluation of ten commercially available cosmetic polymeric microneedle patches (needle heights 180–500 μm, comparable to the MPVA arrays reported here at 319–396 μm) demonstrated that while most commercial products showed acceptable parafilm insertion, only seven of ten patches achieved confirmed ex vivo porcine skin penetration by optical coherence tomography, highlighting that commercial availability does not guarantee consistent insertion performance.^32^ The MPVA-50 arrays fabricated in this study, achieving 84.7 ± 4.2% penetration efficiency at layer 3 (∼381 μm), fall within the performance range of these commercial benchmarks despite being a novel, custom-synthesized hydrogel formulation.

The parafilm insertion data supports the MPVA-50 as the optimal fabrication condition, offering a balance between reliable penetration within the target tissue depth range, controlled puncture geometry, and acceptable interneedle consistency. It is acknowledged that manual thumb pressure introduces variability in load distribution across the array, and future work will employ controlled-force application via a texture analyzer to more precisely characterize insertion mechanics and fracture behavior.

The degree of methacrylation (DM) is a critical parameter influencing the physicochemical properties and processability of PVA hydrogels. Existing literature often reports relatively high degrees of substitution, such as the ∼15% DM observed by.^18^ Similarly, Cavalieri et al. (2004) synthesized PVA-MA derivatives with DMs ranging from 1.0% to 16.8%, noting that while low-modification variants retained water solubility, derivatives with higher substitution required processing in dimethyl sulfoxide (DMSO) due to increased hydrophobicity.^33^ Consistent with these findings, the present study demonstrates that a modest methacrylation level of 3.2% is sufficient to form stable MPVA microneedle structures. While higher degrees of substitution might theoretically yield sharper features, they risk compromising the hydrogel’s integrity by inducing excessive brittleness and rigidity.

Regarding fabrication methodologies, conventional micro molding has historically demonstrated the capability to produce PVA microneedles with superior tip sharpness and mechanical strength.^13^ However, this approach is constrained by the high cost and fabrication complexity of master molds. In contrast, vat photopolymerization circumvents these tooling requirements, offering unparalleled design flexibility and the potential for rapid customization in personalized medicine. Although other photocurable materials, such as GelMA, have achieved greater vertical dimensions in vat photopolymerization processes—reaching heights of 488.1 ± 2.9 μm,^9^ the MPVA MNs optimized in this work provide adequate height for effective transdermal application, balancing geometric sufficiency with the material benefits of PVA.^2^

Among recently reported research-grade hydrogel microneedle systems, GelMA/PVA composite arrays achieved a maximum parafilm penetration depth of 381 μm under manual application, equivalent to the layer 3 threshold demonstrated by MPVA-50 in the present study.^34^ Similarly, 3D-printed stereolithography (SLA) hollow microneedle arrays reported approximately 90% needle penetration to 381 μm with manual thumb application.^35^ The MPVA-50 reported in this study (84.7 ± 4.2% at 381 μm, manual application) is therefore broadly consistent with these studies. It is acknowledged that direct quantitative comparison is limited by methodological differences between studies, including application force, nevertheless, these comparisons collectively indicate that the vat photopolymerized MPVA-50 microneedle arrays achieve insertion performance competitive with commercial products.

## 4 Conclusion

In this work, we used vat photopolymerization 3D printing technology and MPVA to successfully create and optimize a manufacturing technique for hydrogel-forming MN arrays. Spectral analysis (H NMR and FT-IR) was used to confirm the synthesis of MPVA and the effective replacement of hydroxyl groups with photo-cross-linkable methacrylate moieties. Using LAP as a photoinitiator allowed for quick polymerization under 405 nm light. The direct relationship between UV exposure duration and the physicochemical characteristics of the printed MPVA MNs is a key discovery of this work. Our analysis of exposure times of 30, 50, and 70 s revealed a basic vat polymerization trade-off: Insufficient cross-linking density due to under-exposure (30 s) produced mechanically weak structures that are prone to buckling during insertion. The degree of conversion and mechanical stiffness were enhanced by overexposure (70 s), but geometric fidelity was lost, resulting in “voxel bleed” and tip blunting that severely hindered skin penetration. MNs with high aspect ratios, sharp edges, and the compressive strength necessary to consistently break the stratum corneum were produced by optimal exposure (50 s), which produced the perfect balance. These mechanical discoveries were further confirmed by thermal analysis using DSC, which revealed a gradual change in thermal transitions toward higher temperatures with increased exposure, suggesting a denser, more stable polymer network.

Our study creates a solid foundation for the production of PVA-based MNs. We have shown that 3D printing can create biofunctional devices that are on par with those manufactured using traditional techniques in terms of resolution and mechanics while providing better customization options by experimentally determining the ideal processing window. In order to assess the release kinetics and in vivo pharmacokinetics of these improved arrays, future research will concentrate on loading them with drugs.
